# Genome-Wide DNA Methylation and Hydroxymethylation Changes Revealed Epigenetic Regulation of Neuromodulation and Myelination in Yak Hypothalamus

**DOI:** 10.3389/fgene.2021.592135

**Published:** 2021-09-27

**Authors:** Zhixin Chai, Zhijuan Wu, Qiumei Ji, Jikun Wang, Jiabo Wang, Hui Wang, Chengfu Zhang, Jincheng Zhong, Jinwei Xin

**Affiliations:** ^1^Key Laboratory of Qinghai-Tibetan Plateau Animal Genetic Resource Reservation and Utilization, Sichuan Province and Ministry of Education, Southwest Minzu University, Chengdu, China; ^2^State Key Laboratory of Hulless Barley and Yak Germplasm Resources and Genetic Improvement, Institute of Animal Science and Veterinary Research, Tibet Academy of Agricultural and Animal Husbandry Sciences, Lhasa, China

**Keywords:** DNA methylation, hydroxymethylation, neuromodulation, myelination, hypothalamus

## Abstract

Both 5-methylcytosine (5mC) and 5-hydroxymethylcytosine (5hmC) are important epigenetic modifications in neurodevelopment. However, there is little research examining the genome-wide patterns of 5mC and 5hmC in brain regions of animals under natural high-altitude conditions. We used oxidative reduced representation bisulfite sequencing (oxRRBS) to determine the 5mC and 5hmC sites in the brain, brainstem, cerebellum, and hypothalamus of yak and cattle. We reported the first map of genome-wide DNA methylation and hydroxymethylation in the brain, brainstem, cerebellum, and hypothalamus of yak (living at high altitudes) and cattle. Overall, we found striking differences in 5mC and 5hmC between the hypothalamus and other brain regions in both yak and cattle. Genome-wide profiling revealed that 5mC level decreased and 5hmC level increased in the hypothalamus than in other regions. Furthermore, we identified differentially methylated regions (DMRs) and differentially hydroxymethylated regions (DhMRs), most of which overlapped with each other. Interestingly, transcriptome results for these brain regions also showed distinctive gene levels in the hypothalamus. Finally, differentially expressed genes (DEGs) regulated by DMRs and DhMRs may play important roles in neuromodulation and myelination. Overall, our results suggested that mediation of 5mC and 5hmC on epigenetic regulation may broadly impact the development of hypothalamus and its biological functions.

## Introduction

DNA methylation (5-methylcytosine, 5mC), catalyzed by DNA methyltransferases (*DNMTs*), plays important roles in the mammalian neuronal system and embryonic and postnatal development (Li et al., 1992; Okano et al., 1999; Jaenisch and Bird, 2003). It has been reported that *DNMT3a* participates in self-renewal of neural stem cell, and loss of *DNMT3a* significantly impairs postnatal neurogenesis (Hirabayashi and Gotoh, 2010; Wu et al., 2010). Depletion of *DNMT1* and *DNMT3a* negatively affected learning, memory capacity, and synaptic plasticity (Feng et al., 2010). Furthermore, pharmacological inhibition of DNMT activity altered the expression of genes involved in short- and long-term memory formation (Halder et al., 2016). The above studies indicate that proper establishment and maintenance of DNA methylation play regulatory roles in neurodevelopmental process and physiological functions of the mammalian brain. The DNA hydroxymethylation [5-hydroxymethylcytosine (5hmC)] is an oxidized derivative of 5mC, which is converted by ten–eleven translocation (TET) proteins. In addition, 5hmC also acts as an intermediate of active DNA demethylation (Guo et al., 2011). Notably, a high level of 5hmC modification is present in neuronal genomes and thus was assumed to play specific functions in the brain (Kriaucionis and Heintz, 2009). Substantial variation of 5hmC levels between different cell types or tissues have been reported in various vertebrates, such as human cerebellum (Wang et al., 2012) and mouse brain (Guo et al., 2011).

Yak (*Bos grunniens*) is a mammal species habituating in high-altitude conditions and has evolved numerous physiological mechanisms to adapt to these tough environments, including higher metabolism rate, stronger capacity of blood oxygen transportation, and acuter sense for environments when compared to other *Bos* species. Yak has been domesticated in the northeastern Qinghai-Tibetan Plateau (QTP) approximately 7,300 years ago and is the most important livestock in QTP. However, little is known about the genome-wide patterns of 5mC and 5hmC modifications in different regions of yak brain.

Yak mainly lives at altitudes from 3,800 to 4,000 m and cattle lives at altitudes from 1,300 to 1,500 m. Although they are different species, both of them belong to the genus *Bos* and should have a high similarity in genome, body structure, and metabolism mechanisms. Comparisons between yak with cattle have been applied as a general strategy to explore specific mechanisms underlying yak adaptation. In the present study, we compared the genome-wide 5mC, 5hmC, and transcriptome profiles in the brain, brainstem, cerebellum, and hypothalamus between yak and cattle. These results aimed to identify the differentially methylated regions (DMRs), differentially hydroxymethylated regions (DhMRs), and their possible regulatory functions in yak brain.

## Materials and Methods

### Animals and Samples

Three 54-month-old females were sampled from each of the following yak and cattle breeds: Riwoqe yak (sampled from private farms in Riwoqe at altitudes of 3,800–4,000 m; an indigenous yak breed distributed in Riwoqe, Tibet, China) and Sanjiang cattle (sampled from private farms in Wenchuan at 1,300–1,500 m; a local cattle breed developed 200 years ago in Wenchuan, Sichuan, China). There is no direct and collateral blood relationship within the last three generations among the animals of each breed. The animals were housed simultaneously and fed the same diets in local private farms. The sampling was performed in October–December of 2016. The mean live weights of yak and cattle were 188.3 and 163.0 kg, respectively. Before slaughtering, the animals were starved for 1 day. To ameliorate suffering, animals were humanely sacrificed using the following procedure: (1) taking showers for the animals with clean warm water close to the body temperature (35–38°C); (2) animals were knocked out by electric shock (120 V DC, 12 s); (3) during the coma, animals were sacrificed by bloodletting from the carotid artery and jugular vein; (4) after dissection, the brain, brainstem, cerebellum, and hypothalamus were rapidly isolated from each animal. From each issue, 1 cm^3^ of samples were collected, snap-frozen in liquid nitrogen, and stored at −80°C until RNA and DNA extraction.

### Reduced Representation Bisulfite Sequencing and Oxidative Reduced Representation Bisulfite Sequencing

Genomic DNA of each sample was isolated using a QIAamp DNA Mini Kit according to the manufacturer’s protocol (QIAGEN, Valencia, CA, United States). DNA quality and concentration were evaluated using the 2100 Bioanalyzer (Agilent Technologies, Santa Clara, CA, United States) and a NanoDrop spectrophotometer (Rockland, DE). DNA from the same brain region from three animals were pooled. One microgram of pooled genomic DNA was digested using the *Msp*I enzyme for 16 h at 37°C. Next, sequencing libraries were constructed as follows. Briefly, purified digested DNA was treated with a mixture of T4 DNA polymerase, Klenow Fragment, and T4 polynucleotide kinase to repair, blunt, and phosphorylate ends. Next, sequencing control DNA included in the TrueMethyl Seq Kit (Cambridge Epigenetix, Cambridge, United Kingdom) was mixed with the blunt DNA. The mixed DNA fragments were subsequently 3′-adenylated using Klenow Fragment (3′-5′ exo-) and ligated to adaptors using T4 DNA Ligase. The adaptors were synthesized using 5′-methylcytosine instead of cytosine. During each step, the DNA was purified using a QIAquick PCR purification kit (Qiagen, Tübingen, Germany) after the reaction. Before oxidation reaction, all the products were purified using a TrueMethyl Seq Kit (Cambridge Epigenetix, Cambridge, United Kingdom) according to the manufacturer’s instructions. After purification, the oxidation reaction was conducted in a reaction volume of 25 μl with 1 μl of the oxidant solution (Cambridge Epigenetix, Cambridge, United Kingdom) to construct oxidative reduced representation bisulfite sequencing (oxRRBS) libraries. Meanwhile, reduced representation bisulfite sequencing (RRBS) libraries were also prepared as the control, in which 1 μl of ultrapure water was used instead of the oxidant solution. Both the libraries were then treatment at 40°C for 30 min using a thermocycler with the lid temperature of 57°C. Thereafter, the reaction mixture was centrifuged at 14,000 × *g* for 10 min and then the supernatant was transferred to a new 0.2-ml PCR tube for further bisulfite treatment. Bisulfite conversion treatment was performed using a TrueMethyl Seq Kit (Cambridge Epigenetix, Cambridge, United Kingdom) according to the manufacturer’s instructions. The final oxRRBS and RRBS libraries were generated by PCR amplification using adapter compatible barcode primers (Indexed adapter, AG ATCGGAAGAGCACACGTCTGAACTCCAGTCAC; Universal adapter, AGATCGGAAGAGCGTCGTGTAGGGAAAGAGTG TA), examined using an Agilent 2100 Bioanalyzer (Agilent Technologies, Santa Clara, CA, United States) and quantified using real-time PCR assays. After clustering the index-coded samples on a cBot Cluster Generation System using a TruSeq PE Cluster Kit v3-cBot-HS (Illumina, San Diego, CA, United States), the libraries were sequenced on an Illumina HiSeq X Ten platform to generate 150-bp paired-end reads.

### Identification of Differentially Methylated Regions and Differentially Hydroxymethylated Regions

After filtering the low-quality reads, the RRBS and oxRRBS sequencing data obtained from yak samples were aligned to the yak reference genome (Ji et al., 2020), and the RRBS and oxRRBS sequencing data of the cattle were aligned to the NCBI *Bos taurus* reference genome (GCF_000003055.6) using BSMAP (Version 2.74) (Xi and Li, 2009). The level of 5mC at specific sites was directly generated from the oxRRBS library, and that of 5hmC was quantified by subtracting oxRRBS-generated profiles from those generated after bisulfite conversion (ΔβBS-oxBS). Differentially methylated or hydroxymethylated regions (DMRs/DhMRs) were identified using metilene (Version 0.2–6) with default mode (annotates DMRs *de novo* without using any prior information on genomic features) (Juhling et al., 2016). The parameters were set as follows: differential methylation β ≥ 10%, two-dimensional Kolmogorov–Smirnov test *p*-value < 0.05, and Mann–Whitney *U* test *p*-value < 0.05. Enrichment of Gene Ontology (GO) and Kyoto Encyclopedia of Genes and Genomes (KEGG) pathways was conducted using WebGestalt (WEB-based Gene SeT AnaLysis Toolkit).

### Total RNA Extraction, Transcriptome Library Preparation, and Sequencing

The TRIzol reagent (Invitrogen, Carlsbad, CA, United States) was used to isolate the total RNA of each sample. The purity, concentration, and integrity of RNA (RIN > 7.0) were checked using a NanoPhotometer spectrophotometer (IMPLEN, Westlake Village, CA, United States), Qubit RNA Assay Kit in Qubit 2.0 Fluorometer (Life Technologies, Carlsbad, CA, United States), and RNA Nano 6,000 Assay Kit on a Bioanalyzer 2,100 System (Agilent Technologies, Santa Clara, CA, United States), respectively. RNA from the same brain region from three animals were pooled. We utilized 3 μg of high-quality pooled RNA as input material to prepare RNA-seq library for each brain tissue. First, we removed ribosomal RNA using an Epicenter Ribo-Zero rRNA Removal Kit (Epicenter, Madison, WI, United States). Second, the rRNA-depleted RNA was used to create sequencing libraries using a NEBNext Ultra Directional RNA Library Prep Kit for Illumina (NEB, Ipswich, MA, United States). Finally, the library products were purified using 1 × Agencourt AMPure XP magnetic beads (Beckman Coulter, Brea, CA, United States) and an Agilent Bioanalyzer 2,100 System (Agilent Technologies, Santa Clara, CA, United States) was employed to assess the library quality. After clustering the index-coded samples on a cBot Cluster Generation System using the TruSeq PE Cluster Kit v3-cBot-HS (Illumina, San Diego, CA, United States), the libraries were sequenced on an Illumina HiSeq X Ten platform to generate 150-bp paired-end reads.

### Estimation of Transcript Abundance and Differentially Expressed Genes

In-house Perl scripts were used to remove reads containing adapters, poly-N, and low-quality reads from the raw data. The Q20, Q30, and GC contents were then calculated. The paired-end clean reads of the yak and cattle samples were mapped to the newly assembled yak reference genome (Ji et al., 2020) and the NCBI *B. taurus* reference genome (GCF_000003055.6), respectively, using the STAR program (v2.5.1b)^1^ (Dobin and Gingeras, 2015). Mapped reads per sample were assembled using the StringTie program (Pertea et al., 2016) and then merged using the Perl scripts to reconstruct a comprehensive transcriptome per sample. The StringTie program (v1.3.5) and edgeR package (v3.34.0) were used to estimate levels of each transcript (Varet et al., 2016). The StringTie program was used to calculate fragments per kilobase of transcript per million read pairs (FPKM). Differentially expressed mRNAs were identified using the DESeq2 package (v1.32.0), with the criteria of log_2_(fold-change) > 1 or < –1 and *p* < 0.05 (Varet et al., 2016). The differentially expressed genes (DEGs) regulated by 5mC or 5hmC were identified if the DMRs or DhMRs were localized to the DEG or its promoter region.

### Quantitative Real-Time PCR Validation of Selected Differentially Expressed Genes

Compared with other brain regions, the top five upregulated and downregulated genes in yak hypothalamus were selected for qPCR to validate the reliability of transcriptome data. For each species, total RNA was extracted from three animals. Primers targeting these selected genes were designed using the Primer Express software (Applied Biosystems, Foster City, CA) as shown in Supplementary Table 13. All samples were analyzed in triplicate for qPCR assays using the Quant Studio3 Real-time PCR instrument (Applied Biosystems). In the 10 μl of reaction, 5 μl of Fast SYBR^®^ Green Master Mix (Applied Biosystems), 0.4 μl of forward primer (10 μmol/L), 0.4 μl of reverse primer (10 μmol/L), 3.2 μl of nuclease-free water, and 1 μl of cDNA template (8 ng/μl) were included. qPCR amplification procedure consisted of preincubation at 95°C for 1 min, 39 cycles of denaturation at 95°C for 30 s, annealing at 59.5°C for 30 s, and extension at 72°C for 30 s. Dissociation curves were obtained to ensure the specificity of primers. The housekeeping gene *GAPDH* (Glyceraldehyde 3-phosphate dehydrogenase) was tested as the internal control in this study. For each sample, the relative levels of genes were calculated using the 2^−ΔΔCt^ method.

## Results

### Profiling of DNA Methylation and Hydroxymethylation in the Brain, Brainstem, Cerebellum, and Hypothalamus of Yak and Cattle

**FIGURE 1:**
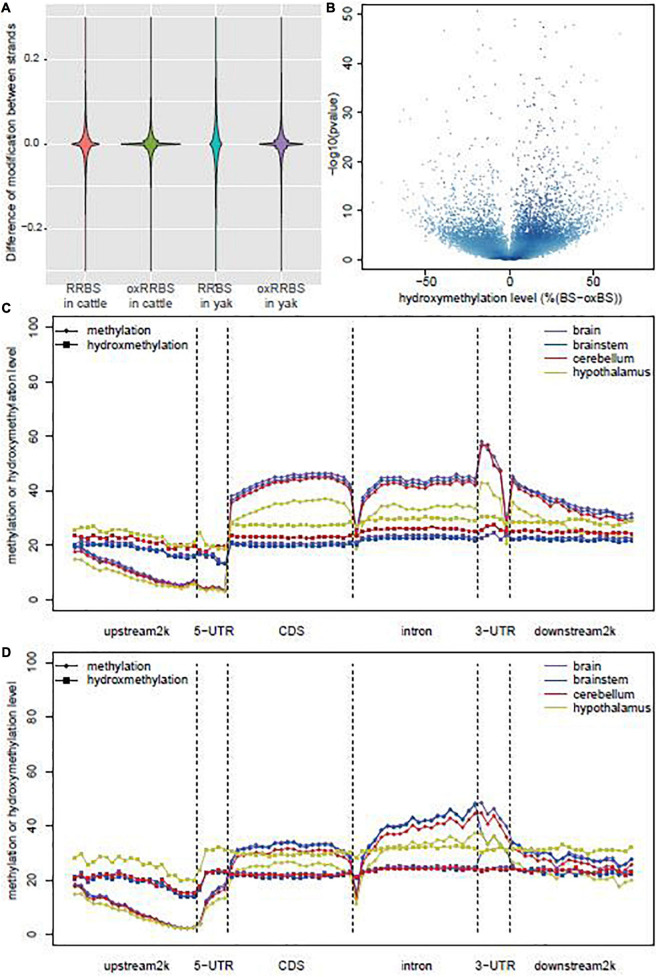
Global DNA methylation and hydroxymethylation among the samples. **(A)** Violin plot of the differences in the modification sites between positive and negative strands in RRBS and oxRRBS libraries. **(B)** Volcano plot of the hydroxymethylation level and the *p*-values were calculated by Fisher’s exact test between RRBS and oxRRBS libraries. **(C)** Distribution of methylation and hydroxymethylation across coding genes in yak. **(D)** Distribution of methylation and hydroxymethylation across coding genes in cattle.

The RRBS and oxRRBS results obtained the single-base resolution of methylation and hydroxymethylation profiling for an average of 3.28M CpG sites with a depth of at least 10 ×. The conversion rates of 5mC and 5hmC in RRBS and oxRRBS libraries were estimated based on the sequencing of the control DNA (Supplementary Table 1 and Supplementary Figure 1). In other mammalian genome, the methylation level of cytosine in the CG sites is highly correlated with the methylation level of the symmetrical cytosine on the opposite strand (Lister et al., 2009). Pairwise correlation analysis of CpGs on the opposite strands within the symmetrical site in each library revealed higher correlation in the oxRRBS library [Pearson’s correlation coefficient (*r*) = 0.82] than that in the RRBS library (Pearson’s *r* = 0.74) (Supplementary Figure 2). Over 82.3% CpGs showed methylation and hydroxymethylation differences within 20% between the opposite strands in the RRBS library, while over 95.4% CpGs showed methylation difference within 20% in the oxRRBS library (Figure 1A). The lower consistency between the opposite strands in the RRBS library might be due to the dynamic 5hmC in the process of active DNA demethylation (Guo et al., 2011).

A previous study reported that a small proportion of CpG sites could be characterized by a negative ΔβBS-oxBS value, possibly generated from technical variance in the sequencing process (Lunnon et al., 2016). We only analyzed CpG sties characterized by “true” levels of 5hmC with the *p*-value of Fisher’s exact test on the difference between RRBS and oxRRBS libraries < 0.05. The distribution of ΔβBS-oxBS values was positively skewed (Figure 1B), and 517,114 and 458,365 loci, on average, were characterized by “true” 5hmC in yak and cattle, respectively. There was no difference in the prevalence of “true” 5hmC sites between yak and cattle. Interestingly, among different tissues, the hypothalamus had the most non-overlap “true” 5hmC sites in yak (235,370) and cattle (165,223), cerebellum had the least non-overlap “true” 5hmC sites (85,778) in yak, and the brainstem had the least non-overlap “true” 5hmC sites (68,918) in cattle. These results suggested the difference in the prevalence of “true” 5hmC sites in the brainstem and cerebellum between yak and cattle (Supplementary Figure 3).

### Levels of 5mC and 5hmC Showed Striking Differences Between the Hypothalamus and Other Brain Regions in Yak and Cattle

The distribution patterns of 5mC and 5hmC levels across genes are shown in Figures 1C,D. 5mC levels across genes were similar to those reported previously with a decrease at the upstream 2 k region, an increase in the coding sequence (CDS) and intron, a sharp decrease at the boundary, and a decrement at the downstream region (Davies et al., 2012; Field et al., 2015). Although the 5mC levels were similar at the upstream region in the brain, brainstem, cerebellum, and hypothalamus, its levels evidently decreased in the hypothalamus at other regions along genes. Meanwhile, 5hmC showed a different distribution pattern in the hypothalamus, with levels consistently higher in the hypothalamus than in other tissues (Figures 1C,D). Interestingly, 5mC level sharply decreased at the boundary between CDS and intron, but 5hmC level did not. These data indicated the different distribution patterns across genes between 5mC and 5hmC, and also striking differences in levels of 5mC and 5hmC between tissues.

**FIGURE 2:**
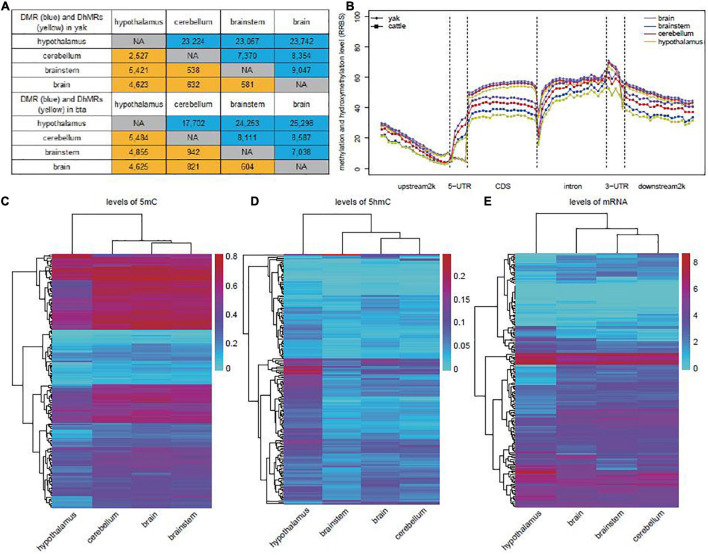
Identification of DMRs and DhMRs in yak and cattle. **(A)** Numbers of DMRs (blue) and DhMRs (yellow) in comparison between each two tissues in yak (top) and cattle (bottom). **(B)** Distribution of total methylation and hydroxymethylation from RRBS library of each tissue in yak and cattle. **(C)** Unsupervised clustering analyses of average methylation levels of yak DMRs related to genes enriched in the nervous system-related GO terms. **(D)** Unsupervised clustering analyses of average hydroxymethylation levels of yak DhMRs associated with genes enriched in the nervous system-related GO terms. **(E)** Unsupervised clustering of mRNA levels of genes associated with DhMRs and enriched in nervous system-related GO terms in yak.

We subsequently identified differentially methylated regions (DMRs) and differentially hydroxymethylated regions (DhMRs) in each pairwise comparison of brain regions between yak and cattle. In yak, the majority of DMRs (73.9%, 70,023 in 94,794) and DhMRs (87.8%, 12,571 in 14,322) were found in the hypothalamus compared to the other brain regions, and the differences were mainly driven by the decreased 5mC level (58,390 of 70,023) and increased 5hmC level (11,920 of 12,571) (Figure 2A and Supplementary Tables 2, 3). Similarly, for the majority of DMRs and DhMRs in cattle hypothalamus, the differences were driven by the decreased 5mC level and increased 5hmC level (Figure 2A and Supplementary Tables 4, 5). We further found that most of the DhMRs (72.9% for yak, 75.7% for cattle) corresponded to the DMRs, suggesting the “waxing and waning” of 5mC and 5hmC levels at the same loci. DNA modification levels from the RRBS library across genes did not reveal any obvious difference among the brain, brainstem, cerebellum, and hypothalamus tissues in yak. However, a striking difference was found among these tissue samples in cattle (Figure 2B). We therefore speculated that the “waxing and waning” of 5mC and 5hmC levels might be a regulatory mechanism in specific tissues or biological processes, and confirmed the necessity of simultaneous investigation of 5mC and 5hmC levels in future studies.

In yak, both DMRs and DhMRs were enriched in the upstream 2,000-bp region from TSS (OR = 1.69, *p* = 3.95e-08), CDS (OR = 3.81, *p* = 3.92e-59), and intron (OR = 4.05, *p* = 1.32e-63) when compared with the location of detectable “CG” in these regions. Similar enrichment patterns were also observed for DMRs and DhMRs in cattle (Supplementary Figure 4).

Enrichment of GO and KEGG pathways was performed for genes related to DMRs or DhMRs in yak and cattle (Supplementary Tables 6, 7). The results showed obvious differences in GO terms between yak and cattle. In yak, the six comparison groups shared four nervous system-related GO terms, which were not enriched in cattle, including “regulation of neurotransmitter levels,” “histamine receptor activity,” “Rho guanyl-nucleotide exchange factor activity,” and “regulation of Rho protein signal transduction” (Supplementary Table 6). Neurons are specialized to receive, process, and transmit information through chemical neurotransmitters between neurons. Once released, neurotransmitters diffuse across the synapse to bind postsynaptic receptors and mediate diverse effects of signaling at the receptor and post-receptor levels (Hyman, 2005). The Gi/o protein-coupled histamine receptor is distributed throughout the CNS including the cerebral cortex, hippocampus, and striatum, and its abundance is the highest in the posterior hypothalamus where the histaminergic cell bodies are located. Histamine receptors are involved in histamine release, impulse flow along the histaminergic neurons, and histamine synthesis. Moreover, they occur as inhibitory presynaptic heteroreceptors on serotoninergic, noradrenergic, dopaminergic, glutamatergic, GABAergic, and perhaps cholinergic neurons (Schlicker and Kathmann, 2017). Rho protein neuron family members are low-molecular-weight guanine nucleotide-binding proteins that function as binary molecular switches by cycling between an active GTP-bound state and an inactive GDP-bound state. Guanine nucleotide exchange factors (GEFs) activate GTPases by enhancing the exchange of bound GDP for GTP. The Rho family of GTPases and related molecules play an important role in various aspects of neuronal development, including neurite outgrowth and differentiation, axon pathfinding, and dendritic spine formation and maintenance (Govek et al., 2005). Unsupervised clustering of levels of 5mC and 5hmC in relation to these GO terms showed distinct modification patterns (Figures 2C,D) and also a distinct mRNA expression pattern of these genes in the yak hypothalamus (Figure 2E).

### Hypothalamus Had a Distinct Gene Expression Pattern Compared With Other Brain Regions in Yak and Cattle

**FIGURE 3:**
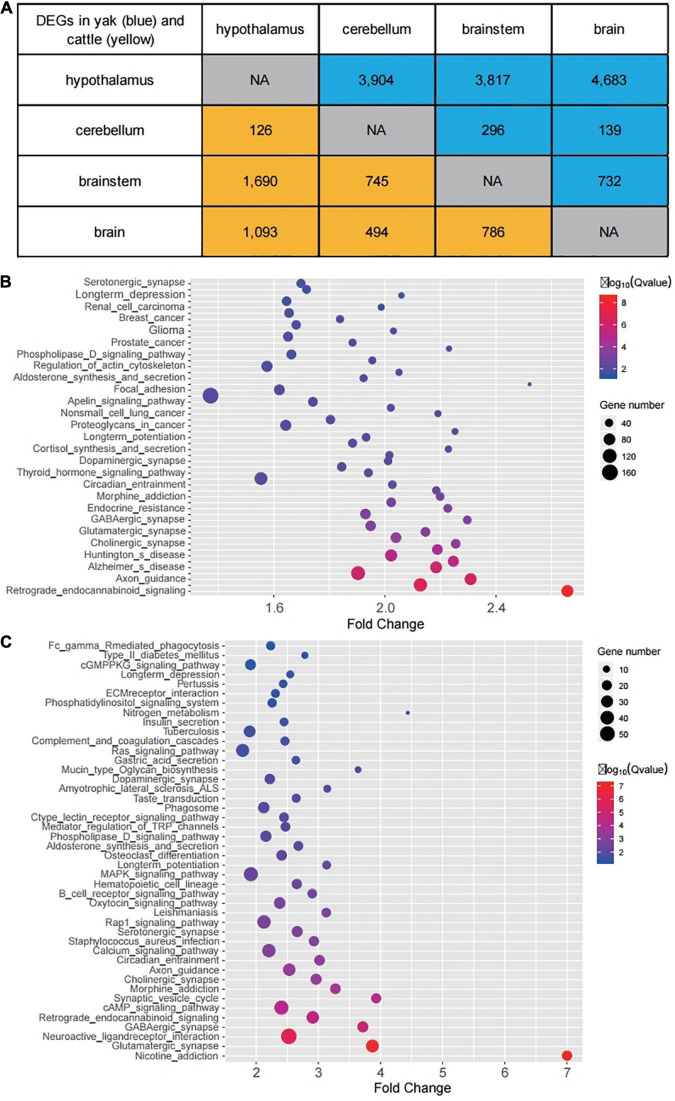
Identification of DEGs in yak and cattle. **(A)** Number of DEGs in comparison between each two tissues in yak (blue) and cattle (yellow). “NA” indicates that comparison analysis is not performed. **(B)** Enriched GO terms and KEGG pathways for DEGs between the hypothalamus and brain in yak. **(C)** Enriched GO terms and KEGG pathways for DEGs between the hypothalamus and brainstem in cattle.

Transcriptome sequencing obtained a total of 22,477 transcripts that were annotated to the GO (Gene Ontology Consortium, 2015), InterPro (Mitchell et al., 2019), KEGG (Kanehisa et al., 2017), Swiss-Prot (Boutet et al., 2016), and TrEMBL (Junker et al., 2000) databases (Supplementary Table 8). The majority of DEGs (91.4%, 12,404 in 13,571) were found in the hypothalamus compared to the other brain regions, and 64.9% (8,044 in 12,404) of these DEGs were downregulated in the hypothalamus (Figure 3A). This result further suggested the “waxing and waning” of 5mC and 5hmC levels, which occurred mainly in the CDS and intron regions, and might play a regulatory role for gene expression. We also observed that the hypothalamus showed the most differences in gene expression when compared with brainstem (*n* = 1,690) and brain (*n* = 1,093) in cattle, which is similar to the changes of 5mC and 5hmC levels in cattle (Figure 3A). However, we found that 55.7% (1,552 of 2,783) of these DEGs upregulated in the hypothalamus compared with brainstem and brain samples, which is more likely regulated by the lowest level of total level of 5mC and 5hmC across genes (Figure 2B). GO and KEGG pathway enrichment was performed for DEGs between different tissues in both yak and cattle. DEGs between the hypothalamus and brain in yak were enriched for nervous system-related terms, such as “retrograde endocannabinoid signaling” (OR = 2.65, FDR = 3.19E-09), “axon guidance” (OR = 2.30, FDR = 3.89E-07), “cholinergic_synapse” (OR = 2.25, FDR = 1.54E-04), “glutamatergic synapse” (OR = 2.14, FDR = 3.41E-04), “GABAergic synapse” (OR = 2.29, FDR = 6.13E-04), “dopaminergic synapse” (OR = 1.84, FDR = 5.62E-03), “neurotrophin signaling pathway” (OR = 1.88, FDR = 5.76E-03), and “longterm potentiation” (OR = 2.25, FDR = 5.88E-03) (Figure 3B). Similarly, DEGs between the hypothalamus and brain in cattle were also enriched for nervous system-related terms, including “glutamatergic synapse” (OR = 3.85, FDR = 8.70E-08), “neuroactive ligand-receptor interaction” (OR = 2.49, FDR = 1.43E-06), “GABAergic synapse” (OR = 3.69, FDR = 1.92E-05), “retrograde endocannabinoid signaling” (OR = 2.88, FDR = 3.46E-05), “synaptic vesicle cycle” (OR = 3.91, FDR = 8.65E-05), “cholinergic synapse” (OR = 2.94, FDR = 3.34E-04), “axon guidance” (OR = 2.51, FDR = 3.75E-04), “serotonergic synapse” (OR = 2.64, FDR = 1.41E-03) and “longterm potentiation” (OR = 3.12, FDR = 4.73E-03) (Figure 3C and Supplementary Tables 9, 10).

### Differentially Expressed Genes Regulated by 5mC or 5hmC in Yak Hypothalamus Played Roles in Neuromodulation and Myelination

**FIGURE 4:**
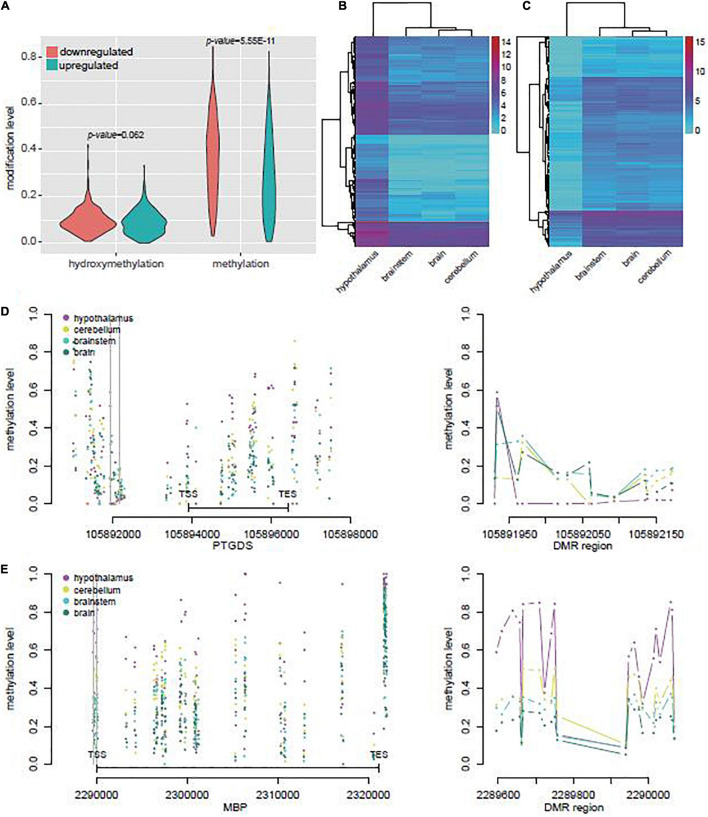
DEGs associated with DMRs or DhMRs in yak. **(A)** Average methylation and hydroxymethylation levels of DEGs associated with DMR or DhMRs, and the DEGs were divided in upregulated or downregulated groups in yak hypothalamus compared with other brain tissues. **(B)** Unsupervised clustering of mRNA levels of the upregulated gene sets in yak hypothalamus compared with other brain tissues. **(C)** Unsupervised clustering of mRNA levels of the downregulated gene sets in yak hypothalamus compared with other brain tissues. **(D)** The methylation levels across the genes and flanking 3,000 bp of the most upregulated gene *PTGS* in yak (left), and the methylation level across one of the DMRs (right), with the location indicated by the gray line on the left. **(E)** The methylation level across the gene body and flanking 3,000 bp of the most downregulated gene *BMP* in yak (left), and the methylation level across one of the DMRs (right), with the location indicated by the gray line on the left.

We identified 652 genes potentially regulated by DMRs or DhMRs and differentially expressed in yak hypothalamus when compared with other brain regions (Supplementary Table 11). In these genes, the upregulated genes had lower average methylation level (Student’s *t*-test, *p* = 5.55E-11) than the downregulated genes in the hypothalamus (Figure 4A). However, both the upregulated and downregulated gene sets revealed similar gene expression levels in the brain, brainstem, and cerebellum (Figures 4B,C), suggesting the distinct gene expression, DNA methylation, and hydroxymethylation profiles in the hypothalamus.

In the top five upregulated genes, *PTGDS* was highly transcribed in the hypothalamus, which might be regulated by the hypoDMR in its promoter (Figure 4D). In the top five downregulated genes, *myelin basic protein* (*MBP*) was highly more abundant in the brain, brainstem, and cerebellum, and might be regulated in hypothalamus by the hyperDMR in its promoter and gene body (Figure 4E).

**FIGURE 5:**
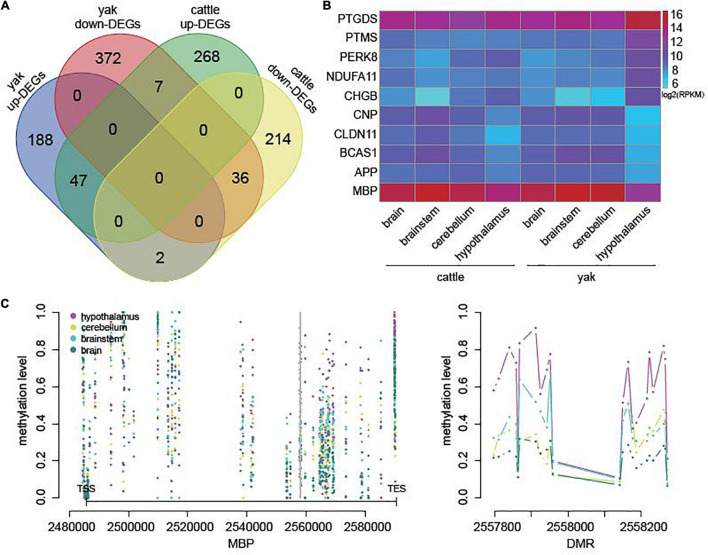
DEGs associated with DMRs or DhMRs in cattle. **(A)** Overlap of DEGs associated with DMRs or DhMRs between cattle and yak. **(B)** The heatmap of mRNA levels of the top five upregulated and downregulated genes in yak hypothalamus compared to the other brain regions. **(C)** The methylation level across the gene body and flanking 3,000 bp of the most downregulated gene *BMP* in cattle (left), and the methylation level across one of the DMRs (right), with the location indicated by the gray line on the left.

In cattle, we also selected 574 genes potentially regulated by DMRs or DhMRs and differentially expressed between hypothalamus and other brain regions (Supplementary Table 12). Among them, only 16.03% were also differentially expressed between hypothalamus and other brain regions in yak (Figure 5A). In addition, compared with other tissues, all the top five downregulated genes in the yak hypothalamus were also downregulated in the cattle hypothalamus, while only one of the top five upregulated genes in the yak hypothalamus were also upregulated in the cattle hypothalamus (Figure 5B). The mRNA level of *MBP* related to myelination might also be regulated by hypermethylation on the gene body in the cattle hypothalamus (Figure 5C).

The mRNA transcription of all the top five upregulated or downregulated genes in yak and cattle brain tissues was validated by qPCR. The results were in accordance with the transcriptome sequencing data (Supplementary Figure 5 and Supplementary Table 13), demonstrating the consistency of epigenetic regulation of myelination in yak and cattle hypothalamus, and distinctive regulation of neuromodulation in the yak hypothalamus.

## Discussion

For a long period, people have known that DNA methylation regulates gene transcription. DNA hydroxymethylation also plays regulatory roles but it is only an intermediate step of the DNA demethylation process (Jaenisch and Bird, 2003; Wang et al., 2012). Previous studies have shown that 5mC and 5hmC levels vary substantially between different cell types and tissues in various vertebrates, and 5hmC is present at relatively high levels in the mature CNS (Kriaucionis and Heintz, 2009), indicating the great involvement of DNA methylation and hydroxymethylation in normal development and function of the mammalian brain. However, there is little research examining the differences in genome-wide patterns of 5mC and 5hmC between multiple brain regions, especially from vertebrates under natural high-altitude conditions. We determined the genome-wide 5mC and 5hmC profiles in the brain, brainstem, cerebellum, and hippocampus tissues using yak as a representative model living in high-altitude conditions and cattle as the control. We applied RRBS and oxRRBS to profile 5mC and 5hmC at the single-base resolution, and our data provided the first map of genome-wide DNA methylation and hydroxymethylation in different brain regions of yak and cattle. The canonical patterns of 5mC and 5hmC levels across the genic regions revealed the unique modification pattern in the hypothalamus, while brain, brainstem, and cerebellum exhibited a similar global distribution of 5mC and 5hmC. These results suggest that both 5mC and 5hmC play important roles in the hypothalamus and might subsequently affect the regulatory functions of hypothalamus in multiple biological processes, such as body temperature, food intake, water balance, blood glucose level, and endocrine gland activity (Burbridge et al., 2016). After the identification of DMRs and DhMRs, we found that DhMR always coexisted with DMRs, suggesting the “waxing and waning” of 5mC and 5hmC levels at the same loci. The RRBS library showed similar DNA modification levels among the brain, brainstem, cerebellum, and hypothalamus in yak, but a striking difference among these tissues in cattle. We speculated that the “waxing and waning” of 5mC and 5hmC levels might be another regulatory mechanism. As another possibility, this difference between yak and cattle might be related to the levels of 5-formylcytosine (5fC) and 5-carboxylcytosine (5caC) (Ito et al., 2011), which were not profiled in this study. Further enrichment analysis showed that genes associated with yak DMRs and DhMRs were involved in the “regulation of neurotransmitter levels,” “histamine receptor activity,” “Rho guanyl-nucleotide exchange factor activity,” and “regulation of Rho protein signal transduction,” indicating the potential roles of DNA methylation and hydroxymethylation in neuromodulation in the yak hypothalamus. Transcriptome sequencing also showed a distinct profile in the yak hypothalamus compared with the other tissues, which is similar to the 5mC and 5hmC profiles. In the yak hypothalamus, the most extensively upregulated gene was *PTGDS*, encoding a glutathione independent prostaglandin D synthase that catalyzes the conversion of prostaglandin H2 (PGH2) to prostaglandin D2 (PGD2). In the present study, transcription of *PTGDS* might be regulated by the hypoDMR in its promoter. PGD2 functions as a neuromodulator and trophic factor in the CNS, involved in smooth muscle contraction/relaxation, and is a potent inhibitor of platelet aggregation (Hiraoka et al., 2001; Huttlin et al., 2017). A recent study revealed that an increase of temperature in preoptic area of the anterior hypothalamus (POA) alters the activity of PTGDS-expressing neurons and then increases PGD2 production. PGD2 activates its receptor DP1 and excites downstream neurons in the ventral medial preoptic area (vMPO) that mediates body temperature decrease, a negative feedback loop for thermoregulation (Wang et al., 2019). However, this negative feedback loop for thermoregulation seems in contradiction with the thermoregulation of yak at long-term low-temperature environments. We speculated that there might be a positive feedback loop for thermoregulation mediated by the expressed PTGDS. *PTMS*-encoded parathymosin may mediate immune function by blocking the effects of prothymosin-alpha that confers resistance to certain opportunistic infections (Martic et al., 2005). *NDUFA11* encodes a subunit of the membrane-bound mitochondrial complex I. Complex I is composed of numerous subunits and functions as the NADH-ubiquinol reductase in the mitochondrial electron transport chain (Stroud et al., 2016). *CHGB* encodes a tyrosine-sulfated secretory protein, which is abundant in peptidergic endocrine cells and neurons, and may serve as a precursor for regulatory peptides (van Vught et al., 2010). *AK1* encodes an adenylate kinase enzyme involved in energy metabolism and homeostasis of cellular adenine nucleotide ratios in different intracellular compartments. *AK1* was reported to highly express in brain (Amiri et al., 2013). These genes might further contribute to physiological adaptabilities to high-altitude conditions. In both yak and cattle, the most extensively downregulated gene (*MBP*) in hypothalamus, which were possibly regulated by hyperDMR in its promoter or gene body, encodes the most abundant protein components of the myelin membrane in the CNS and plays a role in the formation and stabilization of myelin membrane (Guerini et al., 2003). The downregulated mRNA level of *MBP* in hypermethylation indicated the epigenetic mechanism underlying the lower myelin density in the hypothalamus than in the brain, brainstem, and cerebellum. *BCAS1* resides in a region at 20q13 that is amplified in a variety of tumor types, and is reportedly required for myelination (Ishimoto et al., 2017). *CNP* (2′,3′-cyclic nucleotide 3′ phosphodiesterase), encoding myelin, is related to glial cell differentiation and RNA metabolism in the myelinating cell, and is the third most abundant protein in CNS (Voineskos et al., 2008). *CLDN11* also encodes a major component of CNS myelin and plays an important role in regulating proliferation and migration of oligodendrocytes (Gow et al., 1999).

Our study provided the first map of genome-wide DNA methylation and hydroxymethylation in the brain, brainstem, cerebellum, and hypothalamus of yak and cattle. We found a striking difference in 5mC and 5hmC levels and gene expression profiles between the hypothalamus and other brain regions in both yak and cattle, and provided an epigenetic mechanism underlying neuromodulation and myelination in yak hypothalamus. This study supported the idea that 5mC- and 5hmC-mediated epigenetic regulation may broadly impact the development of hypothalamus and its biological functions, which may contribute to physiological adaptabilities to high-altitude conditions.

## Data Availability Statement

The DNA methylation data and RNA transcriptome data in this study is available in SRA under the accession numbers PRJNA602582.

## Ethics Statement

The animal study was reviewed and approved by the Ethics Committee at Institute of Animal Science and Veterinary, Tibet Academy of Agricultural and Animal Husbandry Sciences. Written informed consent was obtained from the owners for the participation of their animals in this study.

## Author Contributions

ZC, ZW, QJ, JX, and JZ planned and coordinated the study and wrote the manuscript. JikW and JiaW collected the samples and performed library construction and sequencing. HW and CZ analyzed the data and assisted in the generation of additional files for the manuscript. All authors read and approved the final manuscript.

## Conflict of Interest

The authors declare that the research was conducted in the absence of any commercial or financial relationships that could be construed as a potential conflict of interest.

## Publisher’s Note

All claims expressed in this article are solely those of the authors and do not necessarily represent those of their affiliated organizations, or those of the publisher, the editors and the reviewers. Any product that may be evaluated in this article, or claim that may be made by its manufacturer, is not guaranteed or endorsed by the publisher.

## Footnotes

1
https://github.com/alexdobin/STAR/releases
