# Supplementary material for: Genome-Wide DNA Methylation and Hydroxymethylation Changes Revealed Epigenetic Regulation of Neuromodulation and Myelination in Yak Hypothalamus
Source: Front Genet. 2021 Sep 27;12:592135. doi: 10.3389/fgene.2021.592135 (PMC8503545; doi:10.3389/fgene.2021.592135)
Supplement: Supplementary file 1 [file Data_Sheet_1.docx]

Supplementary data


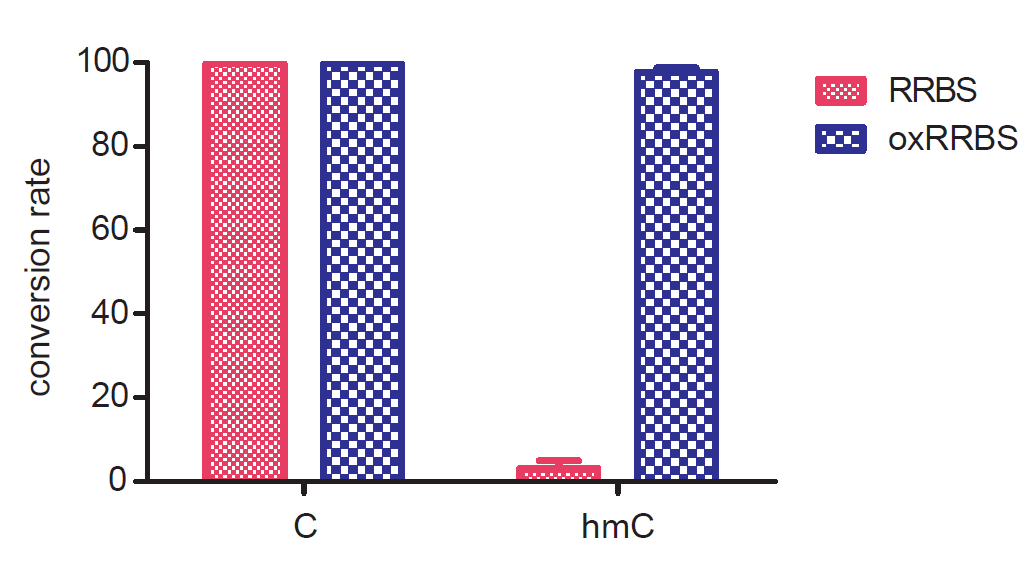


Supplementary Figure 1. The conversion rates of 5C and 5hmC for both RRBS and oxRRBS libraries.


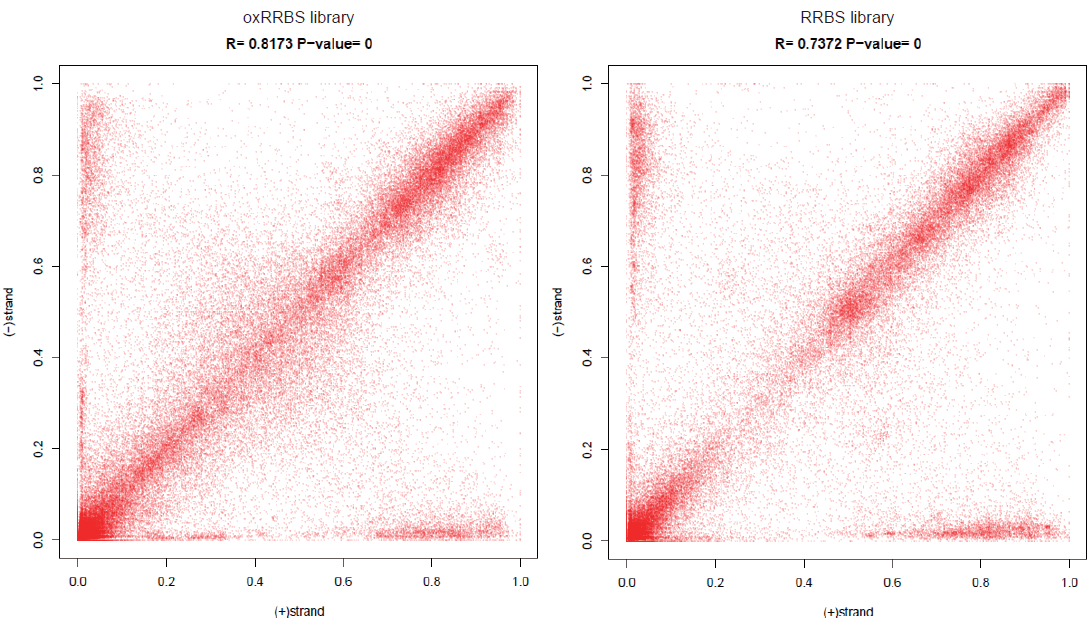


Supplementary Figure 2. Scatter plot of modification level of CpGs on the opposite strands within the symmetrical site in oxRRBS and RRBS library. The correlation coefficients and p-value from Pearson’s test were indicated on the top.


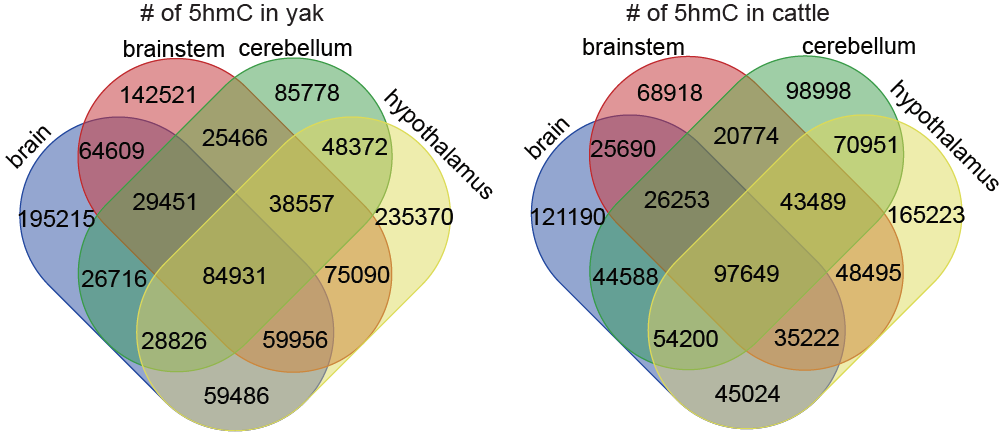


Supplementary Figure 3. Overlap of 5hmC among brain, brainstem, cerebellum and hypothalamus in yak or cattle.


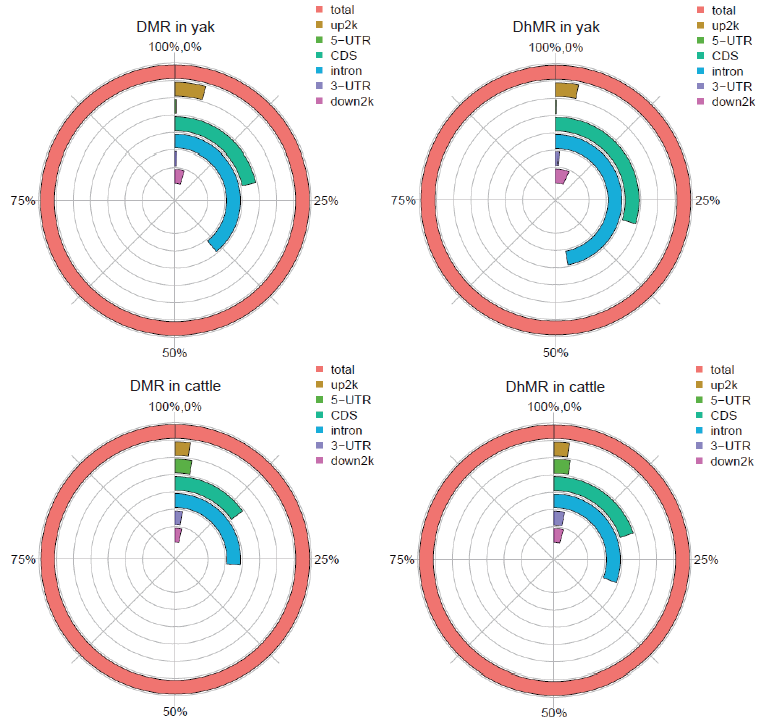


Supplementary Figure 4. Genomic distribution of DMRs (left) and DhMRs (right) in yak (top) and cattle (bottom).


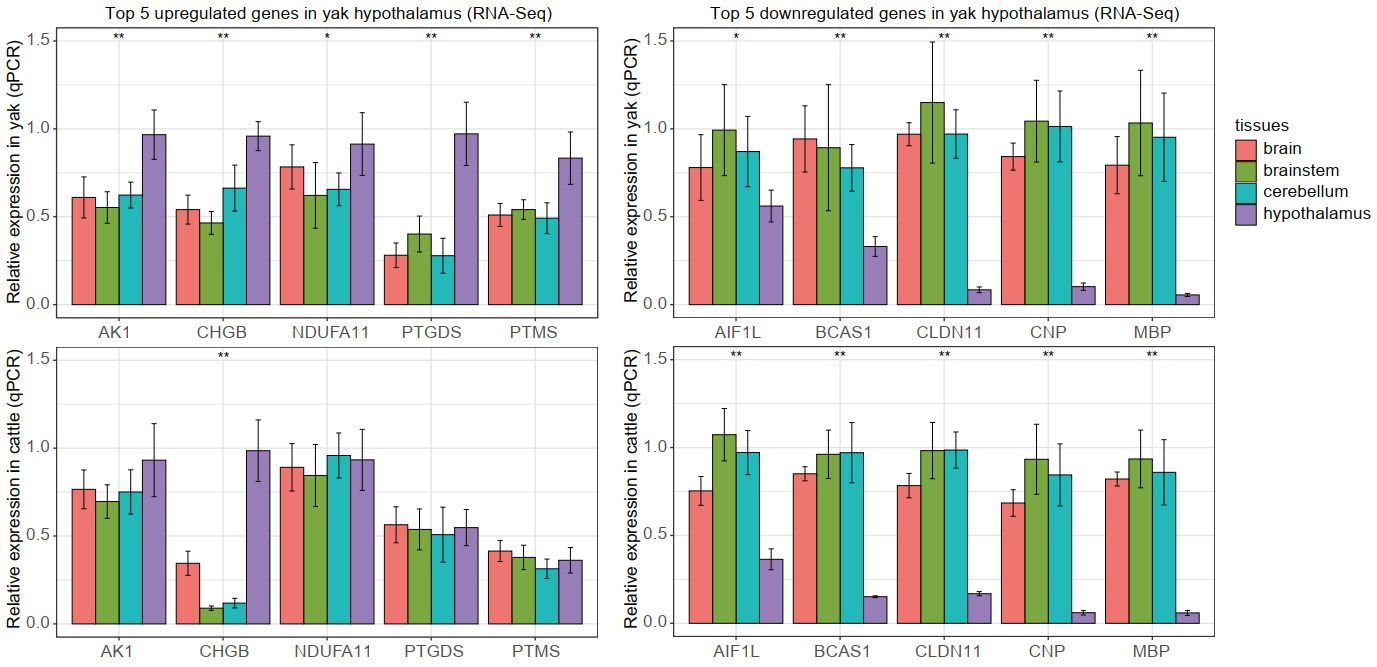


Supplementary Figure 5. qPCR validation for the top 5 upreglated genes in yak hypothalamus (left), and top 5 downregulated genes in yak hypothalamus (right). Relative expression of each gene was determined in each tissue from three yaks or cattles. Data are shown as means±SD (n=3). The p-value of student's t-test for comparison of relative expression between hypothalamus and other brain was indicated at the top (*: p-value <0.05, **: p-value <0.01)
